# MEMS-Based Wavelength-Selective Bolometers

**DOI:** 10.3390/mi10060416

**Published:** 2019-06-21

**Authors:** Thang Duy Dao, Anh Tung Doan, Satoshi Ishii, Takahiro Yokoyama, Handegård Sele Ørjan, Dang Hai Ngo, Tomoko Ohki, Akihiko Ohi, Yoshiki Wada, Chisato Niikura, Shinsuke Miyajima, Toshihide Nabatame, Tadaaki Nagao

**Affiliations:** 1 International Center for Materials Nanoarchitectonics (MANA), National Institute for Materials Science (NIMS), 1-1 Namiki, Tsukuba, Ibaraki 305-0044, Japan; doan.tunganh@nims.go.jp (A.T.D.); sishii@nims.go.jp (S.I.); yokoyamatakahiro1007@gmail.com (T.Y.); handegard.orjansele@nims.go.jp (H.S.Ø.); ngo.haidang@nims.go.jp (D.H.N.); 2 Department of Condensed Matter Physics, Graduate School of Science, Hokkaido University, Kita-10 Nishi-8, Kita-ku, Sapporo 060-0810, Japan; 3 Nanotechnology Innovation Station, National Institute for Materials Science (NIMS), 1-1 Namiki, Tsukuba, Ibaraki 305-0044, Japan; ohki.tomoko@nims.go.jp (T.O.); ohi.akihiko@nims.go.jp (A.O.); nabatame.toshihide@nims.go.jp (T.N.); 4 Research Center for Functional Materials, National Institute for Materials Science (NIMS), 1-1 Namiki, Tsukuba, Ibaraki 305-0044, Japan; wada.yoshiki@nims.go.jp; 5 Center for Green Research on Energy and Environmental Materials, National Institute for Materials Science (NIMS), 1-1 Namiki, Tsukuba, Ibaraki 305-0044, Japan; niikura.chisato@nims.go.jp; 6 School of Engineering, Tokyo Institute of Technology, 2-12-1 Ookayama, Meguro-ku, Tokyo 152-8550 Japan; miyajima.s.aa@m.titech.ac.jp

**Keywords:** wavelength-selective sensors, infrared sensors, perfect absorbers, amorphous silicon, bolometers, microelectromechanical systems (MEMS)

## Abstract

We propose and experimentally demonstrate a compact design for membrane-supported wavelength-selective infrared (IR) bolometers. The proposed bolometer device is composed of wavelength-selective absorbers functioning as the efficient spectroscopic IR light-to-heat transducers that make the amorphous silicon (a-Si) bolometers respond at the desired resonance wavelengths. The proposed devices with specific resonances are first numerically simulated to obtain the optimal geometrical parameters and then experimentally realized. The fabricated devices exhibit a wide resonance tunability in the mid-wavelength IR atmospheric window by changing the size of the resonator of the devices. The measured spectral response of the fabricated device wholly follows the pre-designed resonance, which obviously evidences that the concept of the proposed wavelength-selective IR bolometers is realizable. The results obtained in this work provide a new solution for on-chip MEMS-based wavelength-selective a-Si bolometers for practical applications in IR spectroscopic devices.

## 1. Introduction

Microelectromechanical systems (MEMS, also known as micromachines) technology has been rapidly growing since the 1970s and early 1980s owing to its versatile application in a broad range of devices such as sensors, actuators, micropower generators and microfluidic systems [1,2,3,4,5,6]. Recently, MEMS have been identified as one of the most promising technologies for the industrial internet of things (IoT) market in the 21st century. Recent advances in photonics and nanofabrication techniques have proved the possibility in controlling optical properties of the photonic and metamaterial structures at the desired characteristics (i.e., transmittance, reflectance, absorptivity or polarization) and at the sub-wavelength partial scales. Merging photonic or metamaterial structures with MEMS has enabled a branch of micromachine system for optics and photonics which is so-called micro-opto-electro-mechanical systems (MOEMS) [7]. MOEMS have had a significant impact in optoelectronics ranging from lighting devices to imaging devices, especially in spectroscopic sensing devices such as wavelength selective infrared (IR) sensors and thermography [8,9].

A typical thermographic camera comprises an array of IR MEMS sensors that are designed in the mid-wavelength IR (MIR) atmospheric window (3 µm–5 µm or 9 µm–14 µm). The sensor is customarily made of a photoconductive layer such as InSb, InGaAs, HgCdTe or constructed as a quantum well IR photodetector (QWIP) [10,11]. Latterly, uncooled IR sensors such as thermopile [12,13] and bolometers (thermistors) including vanadium oxide (VO_x_) [14,15,16] and amorphous silicon (a-Si) [17,18,19,20] have emerged as new alternative materials of choice for thermographic devices (microbolometers) because they do not require expensive cooling methods. Especially a-Si has shown great compatibility with the complementary metal–oxide–semiconductor (CMOS) and MEMS technologies [17,19,20].

The common pixel architecture of the microbolometer shows a resonant cavity constructed by a vertical standing wave Fabry–Pérot resonator that enhances the absorption of the incident IR radiation, which is typically designed in the range between 9 µm–14 µm. The resonance of the microbolometer can be further treated at the specific wavelengths to advantageously extend its applications to the multi-spectra IR chemical and medical imaging, IR remote sensing, and non-dispersive IR (NDIR) sensors. In this regard, multi-wavelength or tunable-wavelength microbolometers are desirable for the smart, multi-purpose and portable devices in practical applications. However, it is challenging to integrate multiple vertical-cavity resonators into a multi-wavelength sensors matrix. Another approach for spectroscopic microbolometers is to adopt lateral resonators such as plasmonic antennas [21,22,23,24,25,26], notably plasmonic perfect absorbers [27,28,29,30,31,32], which can energetically absorb and convert IR light into heat to induce the resonant response of bolometers. Furthermore, the lateral resonators in plasmonic perfect absorbers can be easily merged into a multi-wavelength sensor matrix.

The purpose of this work is to investigate the possibility to combine the lateral resonator perfect absorbers with simple MEMS-based bolometers for wavelength-selective IR sensors and multi-wavelength microbolometers. Here the proposed perfect absorber is constructed by a metal–insulator–metal configuration with the top metallic antennas array functioning as a plasmonic resonator [33,34,35]. This perfect absorber configuration has shown great potential in IR photonics because of its simple structure, wide working angle, polarization independence and wide resonance tunability. In particular, this perfect absorber can be easily arranged onto bolometers for wavelength-selective IR sensors. In this bolometer device, the efficient resonant IR energy absorbed in the absorber is directly converted into heat at the metals through Joule heating that follows Poynting’s theorem; then conductively transfers to the bolometer which is thermally isolated from the surrounding media by a Si_3_N_4_ membrane. The reflectance spectra of the fabricated devices matched well with the pre-designed simulation performances. We experimentally showed that the resonance of the bolometer can be readily tuned just by changing the size of the plasmonic resonator. The measured spectral response of a typical device clearly showed the peak matching with the desired resonance. Therefore, the proposed wavelength-selective bolometers are feasible for the practical applications in multi-wavelength microbolometers and spectroscopic IR imaging devices.

The remainder of this paper is organized as follows. Section 2 describes the materials and methods used in this study. Section 3 presents the main results of this work specified in the following sub-sections. Section 3.1 discusses the details of the proposed membrane-supported wavelength-selective bolometers and its optical properties. Section 3.2 shows the fabrication of the a-Si and a-SiGe alloys for bolometers. The detailed fabrication process to realize the proposed bolometers is provided in Section 3.3. Then Section 3.4 shows the resonance tunability of the fabricated bolometers. As a proof of concept, the wavelength-selective responsivity of a typical fabricated bolometer is demonstrated in Section 3.5. A short discussion on the potential applications of the proposed wavelength-selective bolometer is given in Section 3.6. Finally, Section 4 concludes the work.

## 2. Materials and Methods

Simulations: To determine the optimal geometrical parameters (i.e., size of square resonators, the periodicity, and the thickness of insulator- and metal films) for the proposed bolometers to have resonances at desirable wavelengths, numerical simulations were performed. Once the optimal geometries were obtained, we proceeded with fabrication to realize the device. Optical spectra including reflectance, transmittance and absorptivity of the proposed wavelength-selective bolometers were numerically simulated using the rigorous coupled-wave analysis (RCWA) method (DiffractMOD, Synopsys’ RSoft). The electromagnetic field distributions and absorption maps of the proposed device were simulated using full-wave simulations based on the finite-difference time-domain (FDTD) method (FullWAVE, Synopsys’ RSoft). The geometry of the device model was constructed to be identical to the design of the wavelength-selective bolometer using a computer-aided design (CAD) layout (RSoft CAD Environment™, Version 2017.09, Ossining, NY, USA). In the simulation, a 5-nm-thick Ti film serving as the adhesive layers was also added between each interface of Au and dielectric films. The dielectric functions of Au, Ti, Si, SiO_2_ and Al_2_O_3_ were taken from literatures [36,37]. The dielectric function of Si_3_N_4_ was from the reference [38]. For the FDTD simulations, periodic boundary conditions were applied to both the *x*- and *y*-directions and perfectly matched layers were applied to the *z*-axis. In all the simulations, the incident electric field propagated along the *z*-axis and oscillated along the *x*-axis, the incident field amplitudes were normalized to unity.

Fabrications: The a-Si films were fabricated using a DC sputtering (sputter i-Miller CFS-4EP-LL, Shibaura, Yokohama, Japan) involving a boron-doped Si target (0.02 Ω∙cm). For the a-SiGe alloys, a co-sputtering deposition was processed by adding a Ge target. The sputtering conditions of a-Si and a-SiGe alloys films were the same as follows; DC power of 100 W, Ar gas flow of 20 sccm, pressure of 0.304 Pa, sample holder rotation speed of 20 rpm and at room temperature. The membrane-supported wavelength-selective bolometers were processed on a 3-inch double side polished Si wafer. Prior to the fabrication of the devices, a 100-nm-thick SiO_2_ layer was formed on both sides of the 3-inch Si wafer by the dry thermal oxidation at 1150 °C. Subsequently, a-350 nm thick Si_3_N_4_ film was sputtered on both sides of the SiO_2_/Si wafer following a DC (200 W) reactive sputtering recipe from a boron-doped Si target in a mixture of Ar/N_2_ (18/10 sccm) gases. The quality (hardness) of the Si_3_N_4_ films was then amended by a rapid thermal annealing (RTA) process in N_2_ atmosphere (heating rate of 5 °C∙s^−1^, keeping constant at 1000 °C for 1 min, then naturally cooling down). The fabrication of the MEMS devices used several steps of lithography processes including the direct laser writing (*µ*PG 101 Heidelberg Instruments, Heidelberg, Germany) and electron beam writing (Elionix, ESL-7500DEX, Tokyo, Japan) combined with electron beam depositions of metals (UEP-300-2C, ULVAC, Yokohama, Japan) and reactive-ion etching (RIE) of Si_3_N_4_ (CHF_3_ gas, Ulvac CE-300I). The elaborate fabrication of the membrane-supported wavelength-selective bolometers is detailed in the next section.

Characterizations: The amorphous structural property of fabricated a-Si films was verified using an X-ray diffractometer (XRD) with the Cu(Kα) line (SmartLab, Rigaku, Tokyo, Japan). The carrier characteristic of the fabricated bolometers was conducted using a Hall measurement system (Toyo Corporation, ResiTest 8400, Tokyo, Japan). The temperature-dependent resistance measurement was carried out using a source meter (Agilent Technologies B1500A, Santa Clara, CA, USA) combined with a temperature-controlled heating stage. The percent errors of all resistance measurements were less than 2%. The morphological characteristic of the fabricated membrane-supported bolometers was investigated using a scanning electron microscope (SEM) (Hitachi SU8230, Tokyo, Japan) operating at an accelerating voltage of 5 kV. The reflectance spectra of the fabricated devices were measured using an FTIR spectrometer (Nicolet iS50R FT-IR Thermo Scientific, Madison, WI, USA) equipped with a liquid N_2_-cooled mercury cadmium telluride (MCT) detector and a KBr beam splitter. Then the absorptivity spectra were calculated by 1–reflectance, since the transmittance through the absorbers is zero with a 100-nm Au film serving as the bottom mirror. For the spectral response measurement, a tunable IR laser system (104 fs, 1 kHz repetition rate, Spectra-Physics) was used as a tunable excitation source [30]. 

## 3. Results and Discussions

### 3.1. Structural Design and Simulated Optical Properties

**Figure 1:**
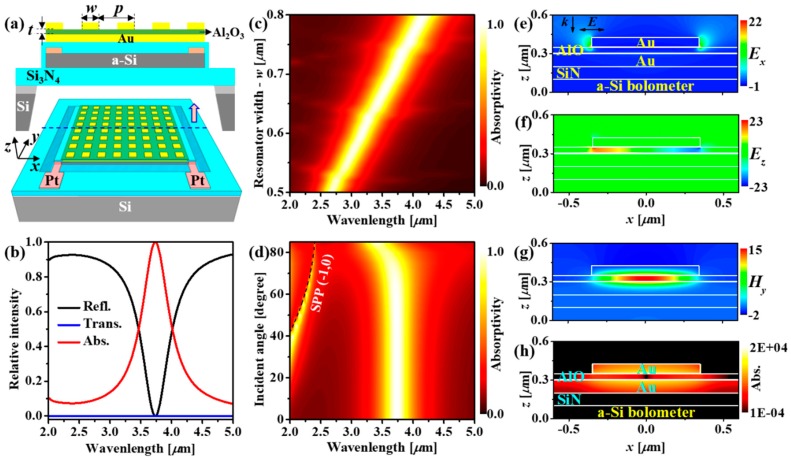
(**a**) Schematic illustrations, tilted view (bottom) and cross-sectional view (top), of the proposed MEMS-based wavelength-selective bolometer. (**b**) Simulated reflectance, transmittance and absorptivity of a device having geometrical parameters of *p* = 1.2 µm, *t* = 0.045 µm and *w* = 0.72 µm exhibits a resonance at 3.73 µm with a unity absorptivity. (**c**) Simulated absorptivity map of the chosen absorber for the proposed device show wide resonance tunability just simply by changing the size of the resonator while keeping other parameters unchanged (*p* = 1.2 µm, *t* = 0.045 µm). (**d**) Simulated angle-dependent absorptivity of the absorber reveals a wide-range working angle up to 70° of the proposed devices. The peak appeared in the shorter wavelength region indicates SPP in periodic Au square array. (**e**–**h**) Simulated electric field (*E_x_*, *E_z_*), magnetic field–*H_y_* and absorption maps of a device excited at the resonance. In the simulations, the incident electric field propagated along the *z*-axis and oscillated along the *x*-axis. The thicknesses of the bottom Au layer and a-Si film were fixed at 0.1 µm and 0.2 µm for all simulations.

Figure 1a shows the proposed MEMS-supported wavelength-selective bolometer. The absorber layer placed on top of the bolometers composes of three layers; a top Au square antennas array serving as plasmonic resonators is isolated from a bottom Au planar mirror via an Al_2_O_3_ dielectric film. The absorbed IR energy in the absorber is converted to heat and then transfers to the a-Si bolometer film. The heat induces the change of the electrical resistance of the bolometer under an external bias applied on the two lateral Pt electrodes. The wavelength-selective absorber is thermally isolated to the Si substrate by a Si_3_N_4_ membrane.

The geometrical parameters of the device including the width of Au square antenna—*w*, the periodicity of the square lattice—*p*, the thickness of the Al_2_O_3_ dielectric—*t* was optimized for certain desirable resonance. The thicknesses of the top Au antenna, the bottom Au film were fixed at 0.07 µm, 0.1 µm, respectively, which are much larger than the skin depth of Au (i.e., 0.03 µm) in the IR region. It is worth noting that the bottom Au film with a 0.1 µm thick is opaque in the MIR region, therefore the optical property of the absorber does not depend on layers underneath. Therefore, the resonance of the absorber relies mainly on *p*, *t* and *w*. Figure 1b plots the simulated reflectance (black curve), transmittance (blue curve) and absorptivity (red curve) of a device having the geometrical parameters of *p* = 1.2 µm, *t* = 0.045 µm and *w* = 0.72 µm. The device exhibits an evident resonance at 3.73 µm with almost unity absorptivity. If the periodicity and insulator layer are unchanged, the resonance wavelength of the device is directly proportional to the width (*w*) of the resonator while it retains perfect absorption in a wide spectral range of the MIR atmospheric window (Figure 1c). This provides a simple way to tune the active wavelength of the bolometer for spectroscopic applications such as for multi-wavelength detection. Further simulations were also performed to verify the effects of other geometrical parameters on optical spectra of the proposed device (Figure S1, Supplementary Information), including the resonator’s thickness (Figure S1a), the insulator’s thickness (Figure S1b), the periodicity (Figure S1c). In particular, with the symmetric resonator (square), the device’s resonance does not depend on the polarization (Figure S1d). The proposed device can be also applied for the near IR and the long-wavelength IR regions (Figure S2, Supplementary Information). The angle-resolved absorptivity was also simulated, and the result is summarized in Figure 1d. Although there exists another resonance in the shorter wavelength region which is attributed to the surface plasmon polariton (SPP) at Au/air interface of the periodic Au square lattice array [38,39], the main resonance (magnetic mode) remains unchanged up to 70° incidence. This large working angle of the proposed device is essential for the practical applications.

To further understand the origin of the perfect resonant absorption in the device, we performed FDTD simulations to calculate electromagnetic field distributions of the absorber used in the proposed bolometer. Figure 1e–g shows the distributions of the electric fields (*E_x_* and *E_z_*) and magnetic field (*H_y_*) of the device excited at the resonance (3.73 µm). In this metal–insulator–metal absorber, the top resonator functions as the sub-wavelength electric dipole antenna which defines the resonance of the absorber. The excited electric field of the electric dipole at the top resonator induces an inverse dipole at the bottom metal film. The oscillation of this antiparallel electric dipoles pair along with the excited electric field creates an electric current loop through the top resonator and bottom metal film, resulting in a large magnetic field enhancement between them (Figure 1g). The resonance of the absorber is so-called magnetic resonance. Interestingly, due to the optical loss of the metal, the absorbed energy at the resonance arises at the top Au resonator and bottom Au film subsequently converts to heat through Joule heating that follows Poynting’s theorem [38,40]. The resonantly generated heat is then conductively transferred to the bolometer film via a thin Si_3_N_4_ layer to trigger the change of the electrical resistance for IR sensing. Here it is worthy that we adopted a 100-nm-thick Si_3_N_4_ layer to electrically isolate the metal film with the bolometer.

### 3.2. Amorphous Silicon and Silicon-Germanium Alloys for Bolometer

Prior to the fabrication of the membrane-supported bolometers, we fabricated bolometer films and examined their temperature coefficient of resistance (TCR) characteristics. In the past two decades, the a-Si and a-SiGe alloys bolometers (thermistors) have been intensively investigated in both scientific study and practical application due to its great success in achieving a high TCR with simple fabrication processes [20,41,42,43]. In this work, we also intended to choose a-Si film fabricated by sputtering for our MEMS-based wavelength-selective bolometers. 

**Figure 2:**
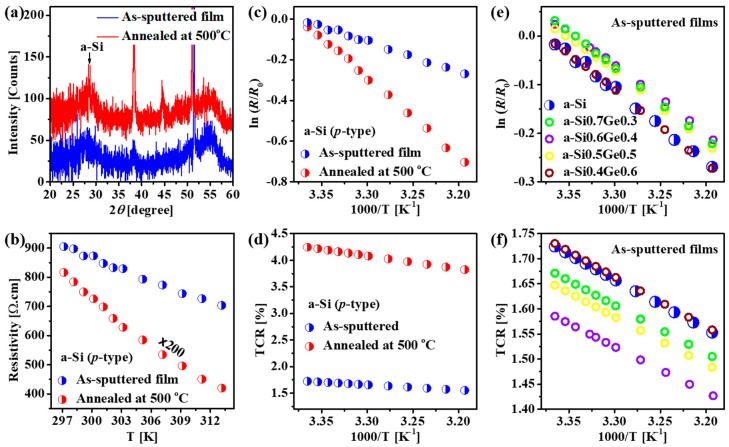
(**a**) XRD patterns and (**b**) Measured temperature-dependent resistivity curves of the as-sputtered (blue curve) and annealed at 500 °C (red curve) a-Si films. (**c**) Natural-log plots represented the change of resistance—$\ln\left(R/R_{0}\right)$ and (**d**) TCR values versus temperature changes of the as-sputtered (blue graphs) and annealed at 500 °C (red graphs) a-Si films. (**e**) Natural-log plots $\ln\left(R/R_{0}\right)$ and (**f**) TCR values versus temperature changes of a variety of amorphous Si-Ge alloys.

Figure 2a displays XRD patterns of two a-Si (*p*-type) films deposited on the 100-nm-thick SiO_2_/Si substrates by DC sputtering using a boron-doped Si target; an as-deposited film shown in blue color and an annealed film for a comparison (500 °C in H_2_ atmosphere) shown in red color. Both two films reveal the amorphous phase of the silicon with two broad 2*θ* peaks at 27.5° and 54° [44]. It is worth noting that the sharp peaks observed in the XRD patters are attributed to the Laue diffraction features of the Si substrate. The carrier characteristics of the as-deposited and annealed a-Si films were carried out via Hall measurements, which indicated that both films showed *p*-type carriers with concentrations of 7.23 × 10^15^ (cm^−3^) and 7.08 × 10^16^ (cm^−3^), respectively. The temperature-dependent resistance property of the a-Si films was performed to evaluate the TCR performance. In the semiconductor, the resistivity is the exponential function of thermal activation conductance expressed by [18]:

(1) $$\rho =\rho_{0}\exp\left(\frac{-E_{a}}{k_{B}T}\right)$$

where ρ, $\rho_{0}$ are the resistivities at a certain temperature and at the initial measured temperature, respectively. $E_{a}$, $k_{B}$ and *T* are the activation energy, Boltzmann constant and temperature (*K*), respectively. Figure 2b presents the temperature-dependent resistivity of the as-deposited and annealed a-Si films. As seen, the resistivities of both the as-deposited and the annealed a-Si films decreases rapidly when the temperature increases. The resistance changes are plotted in $\ln\left(R/R_{0}\right)$, where R and $R_{0}$ are the resistances at temperature *T* and at the initial temperature, respectively, are inversely proportional to the increase of temperature (Figure 2c). The activation energies ($E_{a}$) of the two a-Si films were then obtained from the slope of $\ln\left(R/R_{0}\right)$ against the temperature changes, and the TCR values (α) were finally calculated using the following relation [18]:

(2) $$\alpha =\frac{1}{\rho }\frac{d\rho }{dT}=\frac{E_{a}}{k_{B}T^{2}}$$

Figure 2d reveals the TCR values as a function of temperature. Although the TCR of the as-deposited a-Si film ~1.5% (K^−1^) is lower compared to that of the 500 °C annealed a-Si film ~4% (K^−1^), however, the value is rather good for bolometers. To be compatible with the fabrication of the elaborate MEMS-based devices, thermal annealing is not desirable. We also investigated a-Si_1-*x*_Ge*_x_* alloys for bolometers with *x* variated from 0.3–0.7 (Figure 2e,f). As seen that the performance of the a-SiGe alloys are not much improved or even become worse compared to that of the pure a-Si film. Therefore, we chose the as-deposited a-Si for our MEMS-based wavelength-selective bolometers.

### 3.3. Fabrication of MEMS-Based Wavelength-Selective Bolometers

**Figure 3:**
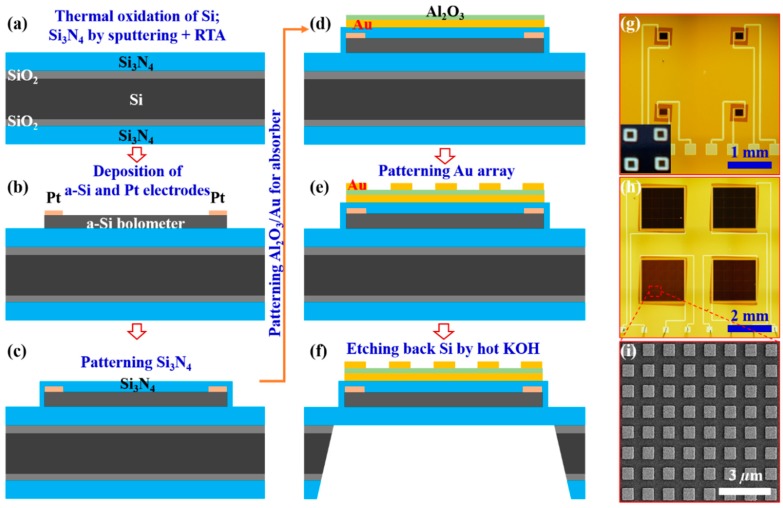
(**a**–**f**) Fabrication procedure of the MEMS-based wavelength-selective absorbers. (**g**,**h**) Top-view optical microscope images of the fabricated MEMS wavelength-selective bolometers with different square antenna sizes of the individual sensors. The inset in (**g**) reveals a photo of the MEMS-based quad-wavelength bolometer with the clear transparent Si_3_N_4_ membrane around each bolometer. (**i**) Top-view SEM image of the typical MEMS sensor having a resonance 3.65 µm.

The MEMS-based wavelength-selective bolometers were then fabricated using several steps of lithography and following the structural parameters optimized by numerical simulations. The fabrication details are shown in Figure 3a–f. The devices were processed on a 3-inch double sides polished Si wafer with Si_3_N_4_/SiO_2_ layered films on both sides (Figure 3a). An array of bolometers with different sizes (0.2 × 0.2 mm^2^, 0.5 × 0.5 mm^2^, 1 × 1 mm^2^ and 2 × 2 mm^2^) and with different wavelength resonances were arranged on the 3-inch wafer. Accordingly, the patterned a-Si films array and their lateral Pt electrodes were fabricated using two steps of lithography combined with sputtering deposition of a-Si and electron beam deposition of Pt (Figure 3b). Subsequently, a patterned 100-nm-thick Si_3_N_4_ layer was conformally deposited on each a-Si bolometer to electrically insulate the bolometer with the Au film of the absorber layer (Figure 3c). Then the wavelength-selective absorber on each bolometer were fabricated depending on the targeted resonance (Figure 3d,e). To process the membrane, a Si_3_N_4_ mask for the anisotropic wet-etching of Si on the back side of each bolometer was formed using RIE (CHF_3_ gas) followed by a photoresist mask. Finally, the membrane-supported Si bolometers array was achieved by applying an anisotropic wet-etching at the back side of the Si wafer using a hot KOH solution (8 mg/l, 80 °C) (Figure 3f). Figure 3g–i explores the structural morphology of the fabricated MEMS-based wavelength-selective bolometers. Figure 3g,h shows typical top-view optical microscope images of the fabricated MEMS sensors, with two types of devices with 0.2 × 0.2 mm^2^ and 2 × 2 mm^2^ active areas. The inset in Figure 3g presents a photo of the MEMS sensors with light illuminated from the bottom which reveals the transparency of the Si_3_N_4_ membrane. Figure 3i presents a top-view SEM image of the fabricated absorber, which clearly shows the square resonator array of the absorber. 

### 3.4. Wavelength Tunability

**Figure 4:**
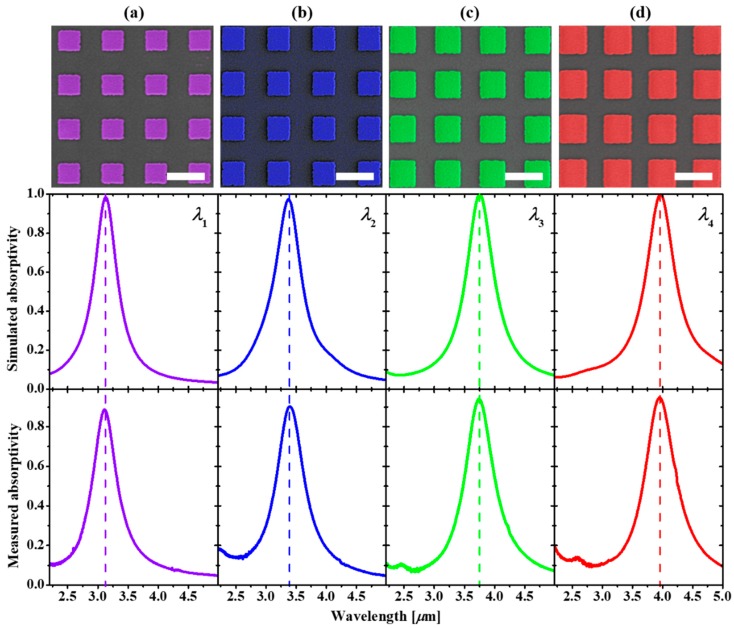
Resonance tunability in the MIR atmospheric window region of the MEMS wavelength-selective bolometers. (**a**–**d**) From top to bottom panels: top-view SEM images (with colors), simulated (middle) and measured (bottom) absorptivities of a quad-wavelength bolometers chip having resonances at 3.11 µm, 3.39 µm, 3.73 µm and 3.96 µm. The scale bar is 1 µm for all SEM images.

The resonance spectra of the bolometers array were firstly characterized using an FTIR. As we discussed, the resonance of the bolometers can be readily tuned just by changing the size of the square resonator. Here we designed and fabricated a series of MEMS-based quad-wavelength bolometers chips. Figure 4a–d, from top to bottom, presents SEM images, simulated and measured absorptivities of the quad-wavelength bolometers. Four absorbers of the device have the same periodicity of 1.2 µm and the same insulator thickness of 0.045 µm but different resonators of 0.59 µm (Figure 4a), 0.64 µm (Figure 4b), 0.72 µm (Figure 4c) and 0.77 µm (Figure 4d) aiming at four different wavelengths of 3.11 µm 3.39 µm, 3.73 µm and 3.96 µm (middle panels of Figure 4). As seen in bottom panels of Figure 4, the fabricated MEMS-based quad-wavelength bolometers chip exhibits near-unity absorption resonances (>0.89) at four different wavelengths which are the same as the pre-designed resonances, evidencing the proper model and precise fabrication process used in this work. The resonances of the MEMS-based quad-wavelength bolometers presented here were chosen in the MIR atmospheric window; they can be also extended to the shorter or longer wavelength regions depending on the specific practical spectroscopic applications.

### 3.5. Wavelength-Selective Responsivity Measurement

**Figure 5:**
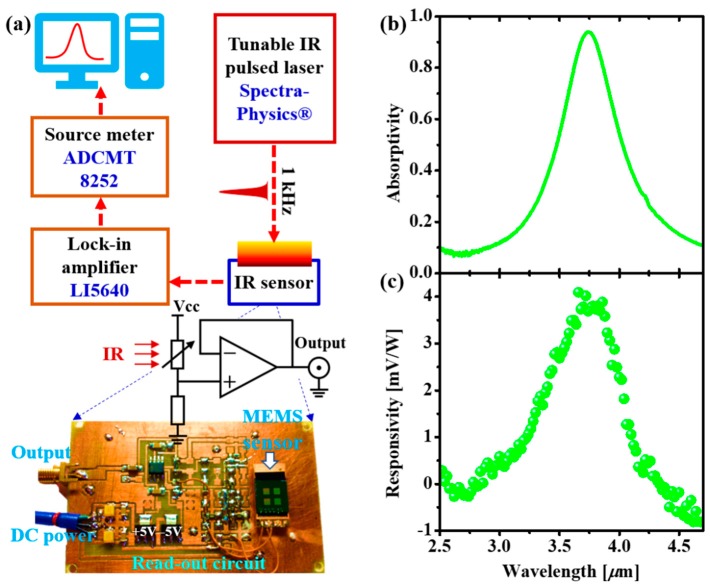
(**a**) Measurement setup of the spectral response of the fabricated bolometers. (**b**) Measured absorptivity and (**c**) Measured responsivity curves of a 3.73 µm resonant bolometer.

To elucidate the spectral response characteristic of the proposed MEMS-based wavelength-selective bolometers, we performed the wavelength-dependent responsivity measurement. The measurement setup is illustrated in Figure 5a. In this measurement, a pulsed tunable IR laser (pulse width of 104 fs, repetition rate of 1 kHz) was used as the excitation source. The IR response of the sensor was measured via a read-out circuit, and then acquired using a source meter collected to a PC. The laser power at each wavelength was above the sensor’s threshold. The spectral response of the sensor was finally normalized to the power spectrum of the laser. 

Figure 5b shows the measured absorptivity spectrum of a typical MEMS-based wavelength-selective bolometer having resonance at 3.73 µm. As we have discussed earlier, the resonantly absorbed IR light at the absorber is converted to heat, then conductively transfers to the bolometer to enable the change of the resistance. The resistance change can be measured as the IR response by applying a current through two Pt electrodes of the bolometer. Figure 5c displays the measured responsivity spectrum of the fabricated device. The measured spectrum responsivity curve exhibits a clear resonance. More interestingly, the resonance peak of the responsivity is the same as the absorptivity peak of the device (i.e., 3.73 µm), proving that the conceptual design for MEMS-based wavelength-selective bolometers is realizable. It should be noted that the measurement was done at a high frequency (1 kHz), because of which the responsivity was just a few mV/W. In the real applications, the device is expected to operate at the lower frequency such as a few hertz, such that the responsivity can be one to two orders higher compared to the value measured at the kilohertz frequency. In addition, the fabricated bolometer presented here have the size of a few hundred microns to millimeter scales; it can be scaled down to a few ten of microns, then the thermal isolation can be further improved for the higher responsivity and response time. The device is also compatible with the bridge-supported sensor technology which is accessible to the read-out integrated circuit (ROIC) for practical applications in IR imaging and thermography.

### 3.6. Potential Applications

Here we discuss some potential applications of the proposed MEMS-based wavelength-selective bolometer. As we mentioned earlier, the proposed device is devoted to serve as a new platform for IR spectroscopic sensors such as portable NDIR sensors, chemical IR imaging or IR remote sensing systems wherein the specific resonances are designed at vibrations of the targeted gases and molecules. The proposed wavelength-selective bolometer can be also applied for compact IR spectrometers, true temperature and emissivity measurements as well as multi-wavelength thermography by integrating multiple resonant sensors in a single chip.

## 4. Conclusions

We have successfully demonstrated a design for the MEMS-based wavelength-selective bolometers. The device used a-Si film deposited by DC sputtering at room temperature as the bolometer material. The fabricated a-Si film showed reasonably high TCR values above 1.5% (K^−1^). A patterned metal–insulator–metal absorber structure was used as the efficient IR light-to-heat transducer for the wavelength-selective bolometers. The proposed bolometers revealed a facile resonance tunability just by changing the size of the patterned square metallic resonators arranged on top of each sensor. We also provided a detailed fabrication procedure to realize the proposed devices. As a proof of concept, we fabricated a set of MEMS-based quad-wavelength wavelength-selective bolometers having resonances in the MIR atmospheric window region. The fabricated devices exhibited the same resonances with the pre-designed devices. More interestingly, the measured spectral responsivity curve showed a clear wavelength-selective response following the absorptivity resonance, which proves the feasibility of the conceptual design of the MEMS-based wavelength-selective bolometers presented in this work. Although this work indicated resonances of bolometers in the MIR atmospheric window region, other resonant wavelengths and different number of resonances can be arranged in a single MEMS-based IR bolometers array chip depending on the specific practical applications such as NDIR sensors, IR spectroscopic sensing or imaging devices.

## Supplementary Materials

The following are available online at https://www.mdpi.com/2072-666X/10/6/416/s1, Figure S1: Effects of the resonator thickness, insulator thickness, periodicity and polarization on optical spectra of the proposed wavelength selective bolometers; Figure S2: Simulated spectra of the proposed device with resonance in the near IR and long-wavelength IR regions.
