# Supplementary material for: MEMS-Based Wavelength-Selective Bolometers
Source: Micromachines (Basel). 2019 Jun 21;10(6):416. doi: 10.3390/mi10060416 (PMC6632019; doi:10.3390/mi10060416)
Supplement: Supplementary file 1 [file micromachines-10-00416-s001.pdf]

Micromachines **2019**, *10*, 416; doi:10.3390/mi10060416

Supplementary Materials: MEMS-Based Wavelength-Selective Bolometers

Thang Duy Dao, Anh Tung Doan, Satoshi Ishii, Takahiro Yokoyama, Handegård Sele Ørjan, Dang Hai Ngo, Tomoko Ohki, Akihiko Ohi, Yoshiki Wada, Chisato Niikura, Shinsuke Miyajima, Toshihide Nabatame and Tadaaki Nagao

Effects of the resonator thickness, the insulator thickness, the periodicity and the polarization on optical spectra of the proposed wavelength selective bolometers.

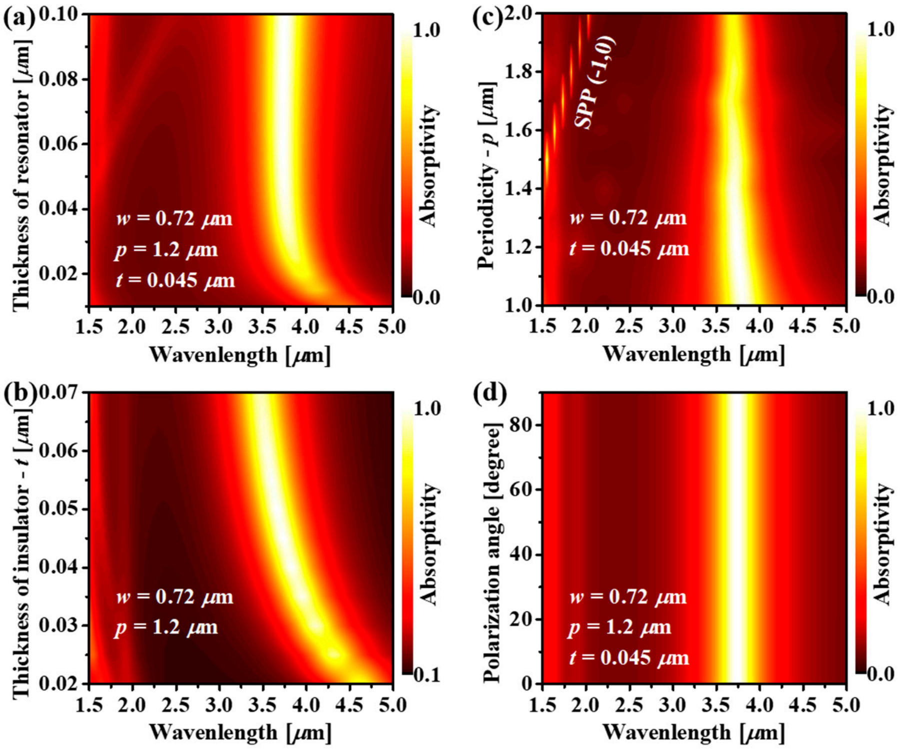

Figure S1. (**a**) Simulated absorptivity map of the proposed wavelength selective bolometer on the resonator thickness. Other geometrical parameters are as follows: resonator width—*w* = 0.72 µm; periodicity—*p* = 1.2 µm; insulator thickness—*t* = 0.045 µm; bottom Au film of 0.1 µm. It is clearly seen that the resonance of the device does not change when the resonator thickness is larger than the skin depth of Au (0.03 µm). (**b**) Simulated absorptivity map of the device having *w* = 0.72 µm and *p* = 1.2 µm while *t* varies from 0.02 µm to 0.07 µm. (**c**) The dependence of the device's resonance on the periodicity while w and *t* are fixed at 0.72 µm and 0.045 µm, respectively. (**d**) Simulated polarization-independent absorptivity of the proposed device (*w* = 0.72 µm, *p* = 1.2 µm, *t* = 0.045 µm).

Simulated spectra of the proposed device with resonance in the near IR and long-wavelength IR regions.

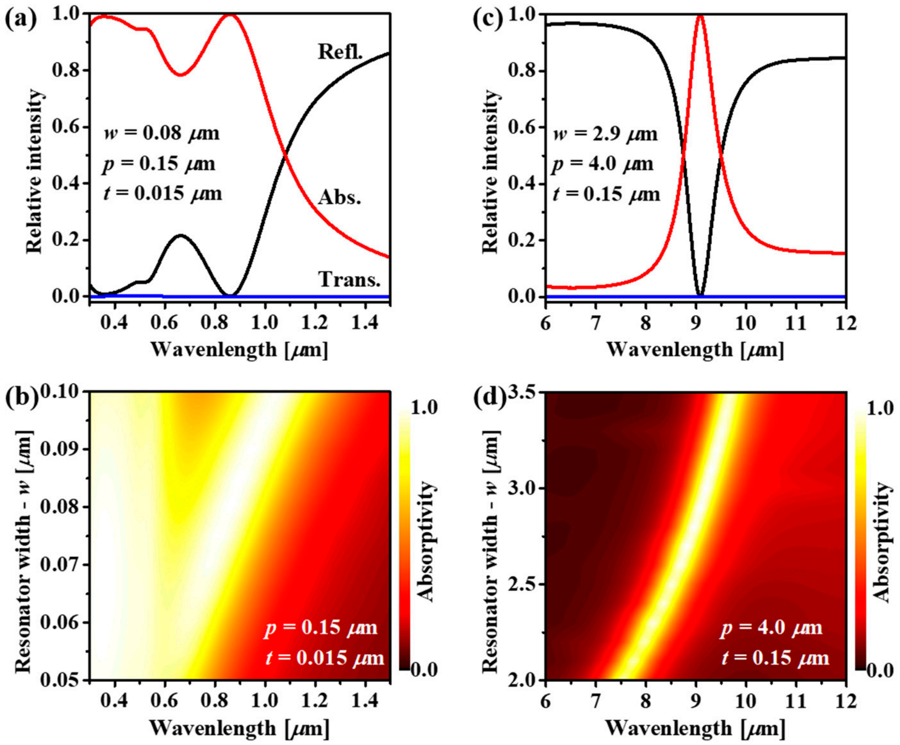

Figure S2. (**a**) Simulated spectra of a wavelength selective bolometer designed in the NIR region (0.86 μm). The geometrical parameters of the absorber are as follows: w = 0.08 μm; p = 0.15 μm; t = 0.015 μm. (**b**) Simulated absorptivity map of the absorber with different resonators while keeping other parameters unchanged (p = 0.15 μm, t = 0.015 μm) shows the resonance tunability in the NIR region. The thicknesses of the square resonator and bottom Au film in simulations of (a) and (b) are 0.04 μm and 0.1 μm, respectively. (**c**) Simulated spectra of a device designed in the long-wavelength IR region (9.1 μm). The geometrical parameters of the absorber are the following: w = 2.9 μm; p = 4.0 μm; t = 0.15 μm. (**d**) The resonance tunability in the long-wavelength IR region by changing the width (w) of the resonator while keeping other parameters unchanged (p = 4.0 μm; t = 0.15 μm). The thicknesses of the square resonator and bottom Au film for all simulations in (c) and (d) are 0.1 μm.
